# Nanoplasmonic Au–Ag Alloy Coatings on the Surface of TiO_2_ Nanotubes for Vitamin B12 Detection by Surface-Enhanced Raman Scattering Spectroscopy

**DOI:** 10.1021/acsomega.5c01060

**Published:** 2025-06-12

**Authors:** Marcin Pisarek, Robert Ambroziak, Mirosław Krawczyk, Marcin Hołdyński, Jan Krajczewski, Tomasz Płociński

**Affiliations:** 1 119463 Institute of Physical Chemistry, Polish Academy of Sciences, Kasprzaka 44/52, Warsaw 01-224, Poland; 2 Faculty of Chemistry, 49605 University of Warsaw, Pasteur 1, Warsaw 02-093, Poland; 3 Faculty of Materials Science and Engineering, 49566 Warsaw University of Technology, Woloska 141, Warsaw 02-507, Poland

## Abstract

The publication shows the possibility of using nano Au–Ag alloy coatings on the surface of TiO_2_ nanotubes to detect vitamin B12. The coatings were prepared using two methods of direct metal deposition: magnetron sputtering and thermal evaporation in UHV conditions. Thanks to the two-stage thermal treatment in a vacuum, Au–Ag alloy layers were obtained: 300 °C/8 h + 450 °C/0.5 h. On these surface-enhanced Raman scattering (SERS) substrates, it was possible to detect vitamin B12 in aqueous solutions at a level of 10^–8^ M. Moreover, the UHV thermally evaporated substrate was characterized by a much better measurement stability for vitamin B12 than the magnetron-sputtered substrate; the relative standard deviations (RSD) were 3.93 and 14.9%, respectively. Based on a less structurally complex probe molecule, 4-mercaptobenzoic acid (PMBA), the tested substrates’ enhancement factors (*E*_F_) were determined as a function of their distribution on the surface. Enhancement maps clearly showed differences in the efficiency of the plasmonic alloy structures obtained, in favor of the samples that are thermally evaporated in UHV conditions, where *E*_F_ changed from 2.3·10^3^ to 5.4·10^4^. The applied methods for depositing Au and Ag metals were crucial in determining the geometric nano factors related to the surface morphology of the obtained layers, which significantly impacted the generation of “hot spots”, where the electromagnetic mechanism (EM) amplification effect occurs most strongly. Detailed SERS measurements and microscopic methods such as scanning electron microscopy (SEM) and scanning transmission electron microscopy (STEM) were used to visualize the surfaces and cross sections of the alloy layers. Other material characterization methods, such as X-ray diffraction (XRD), energy dispersive X-ray spectroscopy (EDX), and X-ray photoelectron spectroscopy (XPS), made it possible to gain an additional information about the structure and chemical composition of the investigated materials. This approach allowed the authors to understand the enhancement effect of the resulting plasmonic structures, in line with the current trend of looking for stable, active SERS platforms with the broadest possible range of applications.

## Graphical abstract

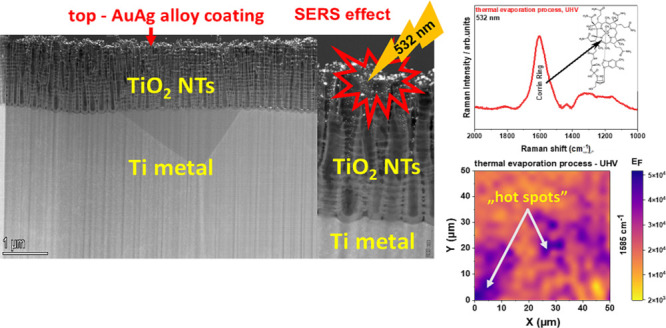

## 1 Introduction

More than 50 years ago, Raman spectra of pyridine were recorded for the first time on an electrochemically roughened silver electrode surface, using the cyclic oxidation and reduction (ORC) method. Fleischmann et al. observed a change in the intensity of pyridine spectra under the influence of excitation with a laser wavelength of 514.5 nm at various silver electrode potentials.^1^ This experiment was related to pyridine adsorption on an Ag surface. Three years later, other researchers took an interest in the observed anomalies in the intensity of the measured spectra of pyridine. Two independent groups, Albrecht and Creighton^2^ and Jeanmaire and Van Duyne,^3^ tried to find the reason for this phenomenon, which had been attributed to the interaction of the probe molecule (pyridine) with the surface of the silver electrode, where an enhancement of the Raman bands of pyridine on Ag compared to the bands originating from free molecules in solution was noted at a level of 10^5^–10^6^. This enhancement was attributed to a surface effect dependent on roughness of the Ag electrode, where the interaction with surface plasmons caused a drastic increase in the Raman scattering cross-section. This led, according to Jeanmaire and Van Duyne^3^ to an increase in the electric field around them, and according to Albrecht and Creighton^2^ to a broadening of the electronic energy levels of pyridine. In 1978, Moskovits noticed that this phenomenon could be effectively used in adsorbate–adsorbent type investigations, where the modified Ag electrode surface showed a collective resonance of pyridine vibration frequency, depending on the degree of its development, in the form of microscale bulges created under the influence of the ORC reaction.^4^ These observations led to the emergence in science of the concept of Surface-Enhanced Raman Scattering (SERS), where the electromagnetic mechanism (EM) played a key role in enhancing the Raman spectra.^4^ In 1981, Pettinger and Wetzel confirmed the research results obtained by the pioneers of the SERS method, using the same method of preparing active substrates on Cu, Ag, and Au based on a cyclic oxidation and reduction process.^5^ Large-scale roughness on the surfaces of the tested electrodes was necessary to obtain intense SER scattering. The enhancement of the recorded spectra of pyridine resulted from the high density of SPP (surface plasmon polaritons) quanta that formed on particularly shaped metal surfaces after the ORC process. Moreover, in this work it is possible to find a relationship between the wavelength of the laser used for excitation and the enhancement of pyridine spectra for Cu, Ag, and Au electrodes. The dependence presented shows that the most active plasmonic metal for SERS applications is Ag, and the weakest is Au, which in practice can be used only in a narrow range of wavelengths. Nevertheless, this profile shows that all three metals cover most of the visible and near-infrared wavelength range, making them convenient.^5^ These wavelength ranges cover most modern Raman measurements.^6^ Further progress related to the development of SERS spectroscopy was made possible by the practical use of an extremely ultrasensitive method often called ″fingerprinting″ in analytical chemistry and elsewhere.^7^ The development of the method could continue due to the use of plasmonic metal nanoparticles and the emergence of computational simulations such as DDA (discrete dipole approximation^8^) or FDTD (finite-difference time-domain^9^), which illustrate the electromagnetic field distribution around materials of various sizes and shapes^10^ in a way unattainable more than 50 years ago. Therefore, researchers are currently focusing on designing new SERS substrates using plasmonic nanoparticles, mainly based on Ag and Au. Cu is characterized by better enhancement than Au, but is a chemically unstable and more reactive metal^5,6,10^ ; this causes significant limitations in many applications where the Cu surface can quickly oxidize.^11^ Unlike Cu, gold is a significantly more chemically stable metal, which is important in environmental studies containing large amounts of water.^7^ We do not observe bands characteristic of water in Raman spectra, as we do in infrared spectroscopy,^12^ and so Au is often used in biological, biomedical, and food control research.^13,14^ Considering the advantages and disadvantages of plasmonic metals such as Cu, Ag, and Au regarding their optical properties, compromise solutions between electromagnetic amplification and the chemical stability of the designed SERS substrates are currently being sought. This feature is significant in bioanalysis, where high substrate stability and measurement repeatability are required, which can be provided by Au–Ag alloy systems.^15^ Moreover, such applications require an appropriately selective substrate, which can be controlled by the size and shape of the nanoparticles.^15–17^ Also important is an appropriate range of laser wavelength (its energy) in terms of the properties of the plasmonic metal and the detection of a specific molecule, which can significantly improve the selectivity of this type of SERS platform, where resonant Raman scattering via a chemical mechanism can play a key role.^15^ Two ways of producing SERS-active substrates are currently being developed: top-down and bottom-up methods.^17–19^ The top-down method of forming platforms is mainly based on lithographic methods and selective chemical etching using special templates.^18,19^ The second, much more popular method involves selected various chemical, electrochemical, and physical methods that cause a nanostructuring of the surface of solid materials.^18,19^ In most cases, the prepared substrates are additionally coated with plasmonic metals.^11,19^ In both methods, the nanostructuring of the substrate is aimed at forming appropriate distances between plasmonic metal nanoparticles of different shapes and sizes (appropriate distribution) or at forming a developed surface that will favor the generation of a strong electromagnetic field in depressions, crevices, and surface roughness. The points that generate a strong electromagnetic field are known as “hot spots”,^18,19^ and their surface population correlates to the enhancement factor of the designed and manufactured SERS platforms.^18,19^ Considering the above guidelines, the authors of this work focused their attention on designing and producing SERS substrates based on nanostructured TiO_2_ oxide with a layer of bimetallic Au–Ag alloy. A bottom-up method was used to form TiO_2_ nanotubes on a Ti substrate. Then, the ordered substrates prepared in this way were functionalized by the deposition of Au and Ag layers of the same thickness, one by one, using the thermal evaporation method or magnetron sputtering. Next, the TiO_2_ NTs/Au–Ag bimetallic layer systems were annealed in two stages under UHV conditions to produce an Au–Ag alloy on the surface of the nanotubes.^20^ The novelty of this research was to determine what topographic and chemical factors influence the enhancement of the Raman signal of the tested analytes, depending on the vacuum conditions of the deposition of the plasmonic metals, which were then heated using the same thermal treatment parameters. This procedure led to the formation of active and effective SERS substrates for detecting vitamin B12 in aqueous solutions, while the high measurement stability of the investigated systems was maintained. This stability was related to the compromised plasmonic properties of the obtained Au–Ag layers on TiO_2_ nanotubes as regards their spatial geometry, which was revealed by high-resolution SEM and STEM microscopic observations. Vitamin B12 is a complex compound in which the central cobalt atom is coordinated with nitrogen atoms conjugated to pyrrole rings, which create a macrocyclic corrin system (which is a reduced form of porphin).^21^ This characteristic structure enables the use of vitamin B12 in SERS research where resonance Raman spectroscopy (RRS) has played an important role in understanding and examining the structures of chemical molecules, including extremely large ones such as vitamin B12 as well as many chemical and biochemical processes involving these molecules.^21^ In living organisms, vitamin B12 acts, among other things, as a regulator of the production of erythrocytes (red blood cells). Vitamin B12 deficiency causes anemia. It is classified as a B vitamin, which are water-soluble precursors of coenzymes.^22,23^ Considering the advantages of SERS spectroscopy, i.e. the possibility of detecting molecules with concentrations below 10^–9^ mol/L using a laser with a wavelength in the visible range, which limits the signal from water and quenches the fluorescence that constitutes the background in classic Raman spectra, this method can be a fast, effective way to analyze systems containing vitamin B12 at various concentrations.^24^ Designing and manufacturing appropriate sensors for the detection of B vitamins, including B12, is an ongoing challenge due to the variety of methods of supplementing these chemical compounds, which are of great importance in food analysis techniques and medicine (*in situ* monitoring of the kinetic transformations of a chemical product).^25,26^ Therefore, finding and developing simple, cheap methods for preparing SERS substrates in this type of application is extremely important.

## 2 Experimental Section

Taking into account the introduction and the aim of the work, Scheme 1 shows the idea of designing and manufacturing SERS active substrates based on TiO_2_ nanotubes with bimetallic Au and Ag layers.

**Scheme 1:**
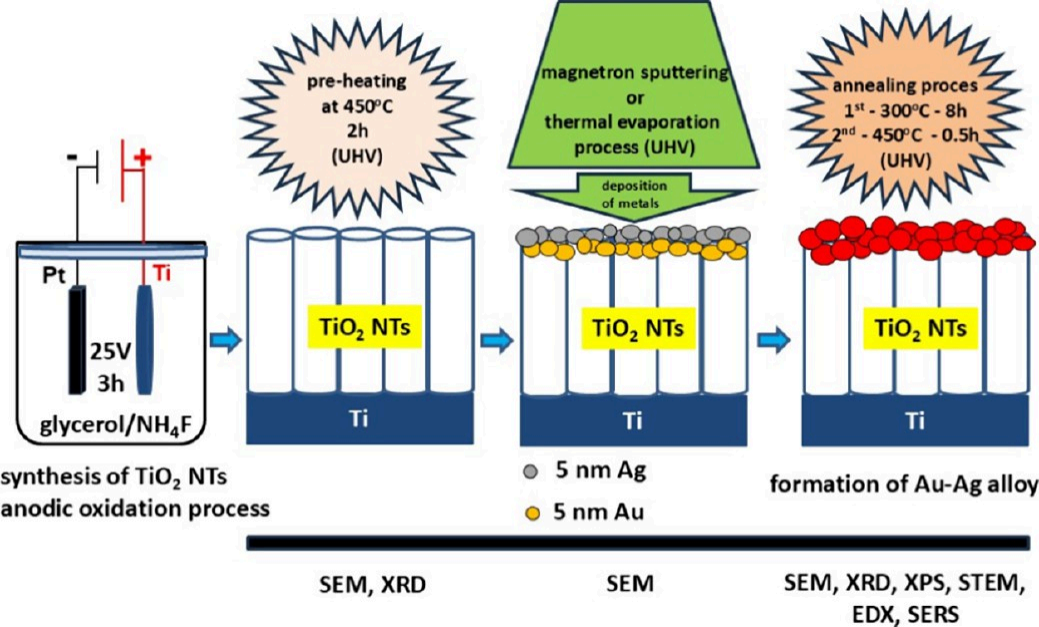
Method of Production and Surface Functionalization of TiO_2_ Nanotubes for SERS Applications^a^

a Step one: synthesis of TiO_2_ nanotubes by anodic oxidation process; step two: initial thermal treatment at 450 °C under UHV conditions/2 h in order to obtain the crystal structure of TiO_2_ in the form of anatase and improve the mechanical stability of the TiO_2_/Ti system; step three: deposition of plasmonic metals Au and Ag in the form of monometallic layers or bimetallic system (Au/Ag) using PVD methods (thermal evaporation in a vacuum or magnetron sputtering); step fourth: two-stage thermal treatment of bimetallic layers under UHV conditions (300 °C for 8 h and 450 °C for 30 min) in order to obtain Au–Ag alloy systems on the top of TiO_2_ nanotubes.

Experimental details are described below.

### 2.1 Formation of TiO_2_ Nanotubes

The synthesis of TiO_2_ nanotubes was carried out based on the anodic oxidation of Ti foil with a purity of 99.5% (Alfa Aesar) at a constant voltage of 25 V/3 h in a two-electrode system, where the working electrode was Ti with an area of 1 cm^2^ and the counter electrode was Pt of the same area. The process was performed in a solution of water and glycerin at a ratio of 50:50, with the addition of ammonium fluoride 0.27 M. The samples were then rinsed in deionized water and air-dried. The prepared substrates were then vacuum preheated at 450 °C for 2 h. This procedure was aimed at obtaining a stable structure of titanium oxide in the form of anatase.

### 2.2 Deposition of Au and Ag Metals

#### 2.2.1 Thermal Evaporation in UHV Conditions

An effusion cell EF40C1 placed in the UHV preparation chamber (PREVAC, Rogów, Poland) was used to deposit monometallic silver (Ag) and gold (Au) layers, both 10 nm in thickness, and a bimetallic layer of Au–Ag, on TiO_2_ nanotubes (NTs) by means of thermal evaporation. An Au (5 nm)-Ag (5 nm) bilayer, with the individual metal layer thicknesses shown inside the parentheses, was fabricated through the two-step sequential deposition of a 5 nm-thick Au layer first, followed by 5 nm of Ag. The Ag (0.1 mm-thick Ag foil, Premion, 99.998% purity, Alfa Aesar) and Au (0.1 mm-thick Au foil, 99.9975+% purity, Alfa Aesar) were deposited at a pressure of (1–2) × 10^–8^ mbar with constant evaporation rates in the range of 0.02–0.03 nm/min, monitored by a quartz crystal thickness monitor TM-400 (Maxtek Inc.). It register changes in the frequency of the quartz crystal placed in the preparation chamber in time during deposition of both metals, and displays the calculated deposition rate and layer thickness for the selected time intervals. These parameters resulting from the quartz crystal microbalance (QCM) technique were then applied to deposit monometallic Ag and Au layers, and a bimetallic layer of Au–Ag on TiO_2_ NTs.

#### 2.2.2 Magnetron Sputtering

Metal layers of silver (Ag) or gold (Au) with a thickness of 10 nm each and a combination of a layer by layer system, where 5 nm of Au was followed by 5 nm of Ag, were deposited by magnetron sputtering using a Leica EM MED020 DC sputtering instrument. During the deposition, silver and gold foils (0.2 mm thick each, Kurt J. Lesker, 99.99% purity) were used as targets. All the deposits were carried out at room pressure, with an applied current of 25 mA and 2 × 10^–2^ mbar Argon pressure. The whole process was controlled with a quartz crystal film thickness monitor (EM QSG100). Before deposition, the samples were put into the 10^–5^ mbar vacuum range, followed by target cleaning with a presputtering step for about 30 s with a closed shutter.

### 2.3 Two-Stage Heat Treatment under UHV Conditions

To effectively alloy the Ag and Au, an Au–Ag bilayer deposited on TiO_2_ NTs was annealed under a 10^–8^ mbar vacuum at 300 °C for 8 h, followed by 30 min at 450 °C.^20^

### 2.4 Material Characterization

#### 2.4.1 Scanning Electron Microscopy and Energy Dispersive X-ray Spectroscopy

Scanning electron microscopy (SEM) images were obtained under a high vacuum (10^–7^ mbar) using a Nova NanoSEM 450 instrument (FEI Company). The images presented were collected with a Through Lens Detector (TLD) of secondary electrons at a primary beam energy of 10 keV and a working distance of 5 mm from the pole piece. After the selection of the inspection region, data were recorded with a long scan acquisition time (20 ms) of, typically, 30 s per frame. Energy dispersive X-ray spectroscopy measurements (EDX) were carried out using an EDAX Octane Elect EDS system with silicon drift detector (SDD) technology. All measurements were performed under the same conditions as the imaging, with an electron beam energy of 10 keV.

#### 2.4.2 X-ray Diffraction

X-ray diffraction data were collected on a PANalytical Empyrean diffractometer fitted with an X’Celerator detector using Ni-filtered Cu Kα radiation (λ_1_ = 1.54056 Å and λ_2_ = 1.54439 Å). Flat plate θ/θ geometry with a spinning sample holder was used to obtain the results (16 s revolution time). All the data were recorded in the 10–90° 2θ range, with steps of 0.017° and a scan time of 20 s per step.

#### 2.4.3 Scanning Transmission Electron Microscopy and Energy Dispersive X-ray Spectroscopy

The observations of the samples were carried out using a High Resolution Scanning Transmission Electron Microscope made by ThermoFisher Scientific, model Spectra 200. A thin sample was prepared by using a Focus Ion Beam (FIB) system made by Hitachi High Technologies, model NB5000. The lift-out technique was used for the sample preparation. The observations were taken at 200 kV in the STEM mode, using BF and HAADF detectors. For the chemical composition analysis, an Energy Dispersive X-ray (EDX) spectrometer was used to collect the data and produce chemical composition maps of Au, Ag, Ti and O, as well as in the quantification analysis. For the quantification we used the Brown-Powell Ionization cross-section model and multipolynomial background correction.

#### 2.4.4 X-ray Photoelectron Spectroscopy: *In Situ* Measurement after the Two-Stage Thermal Treatment

After completion of the two-stage thermal treatment of TiO_2_ NTs coated with bimetallic Ag–Au deposits, the samples were transferred *in situ* into a PHI 5000 VersaProbe XPS Microprobe (ULVAC-PHI, 2500 Hagisono, Chigasaki, Kanagawa, Japan). The XPS measurements were carried out using microfocused and monochromatic Al K_α_ radiation (*h*ν = 1486.6 eV) from an X-ray source operating at a 100 μm spot size of 100 μm, at 25 W and 15 kV. The analyzed area was defined as 500 μm square. High-resolution (HR) XPS spectra of Ti 2p, O 1s, Ag 3d, Au 4f, C 1s and valence band (VB) signals from the samples were collected with a hemispherical analyzer at a pass energy of 23.5 and an energy step size of 0.1 eV. The X-ray beam was incident at the sample surface at an angle of 45° with respect to the surface normal, and the analyzer axis was located at 45° with respect to the surface. XPS data analyses were performed using Avantage Surface Chemical Analysis software (ThermoFisher Scientific, ver. 5.9911). To deconvolute the HR XPS spectra, we applied a smart-type background and a Gaussian peak shape with a 35% Lorentzian character. The binding energies (BEs) of all the detected elements were corrected with respect to the BE of the C 1s peak at 284.8 eV on gold reference sample.

### 2.5 Spectroscopic Characteristics

#### 2.5.1 Surface-Enhanced Raman Scattering Measurements

The SERS Raman spectra were collected with a Horiba Jobin-Yvon Labram HR800 spectrometer equipped with a Peltier-cooled CCD detector (1024 × 256 pixels), a 600 groove/mm holographic grating, and an Olympus BX40 microscope with a long distance 50× objective. A diode-pumped, frequency-doubled Nd:YAG (532 nm) laser provided the excitation radiation for the vitamin B12 and PMBA measurements. The 4-mercaptobenzoic acid samples were prepared by immersing the substrate in a saturated PMBA solution for 24 h, then rinsing it with water and allowing it to dry for 1 h. In the case of vitamin B12, various aqueous solutions were prepared in a concentration range of from 10^–5^ M to 10^–8^ M. Then, 50 μL of the selected solution was applied to the sample and the measurement was performed through the droplet after focusing the laser beam on the prepared SERS substrates. All spectra presented in this study represent an average of 400 spectra recorded over an area of 50 × 50 μm. Based on the spectra recorded in this way, a map of the enhancement factor distribution for the PMBA acid molecule at a shift of 1590 cm^–1^ was generated by calculating *E*_F_ point by point.

## 3 Research Results and Discussion

Figure 1 shows SEM images of the surface of the fabricated materials after various metal deposition processes in a vacuum. The first series of samples (Figure[Fig fig1]a,b) shows the Ag deposit. Differences in the distribution of Ag on the tops and walls of TiO_2_ nanotubes can be observed. After thermal evaporation of Ag under UHV conditions (10^–8^ mbar), individual nanoparticles or their agglomerates can be seen to decorate the surface of the nanotubes. In the case of magnetron sputtering (10^–2^ mbar), Ag is also located on the tops of the nanotubes, but forms rings with a less developed surface area than using the evaporation method. The same phenomenon can be seen for the series of samples with an Au layer. Nevertheless, for these substrates, the distribution of Au appears to be more homogeneous, as does the size of the nanoparticles (see Figure 1c). In contrast, the rings that form around tubes correlate more with their size and wall thickness (see Figure 1d). Our previous research has shown that, at a voltage of 25 V and a time of 3 h, nanotubes with an average diameter of about 110 nm and a wall thickness of about 20 nm are formed.^27^ After the annealing process, for the samples with an Au/Ag deposit, layer by layer, apparent differences in the surface morphology can also be seen, depending on the metal sputtering method used. For the thermal evaporation method under UHV conditions (Figure 1e), we still observe the effect of nanoparticles around the nanotubes, with larger differences in their size for pure Au and Ag. In addition, the nanoparticles have a more spherical/homogeneous shape, being a result of the annealing process. In the case of the magnetron-sputtered samples layer by layer (Figure 1f), we observe a completely different surface morphology. The characteristic rings are missing from the SEM images. Relatively large objects with irregular shapes, and more spherical objects of smaller size, are visible. This distribution of metals is probably the result of their “melting” on the surface of the nanotubes. The different surface morphology of the substrates after annealing processes under UHV conditions may indicate that two materials have been obtained for SERS applications, with various distributions and shapes of plasmonic metals.^11^ Such a morphology with a highly ordered degree of surface development thanks to the use of TiO_2_ nanotubes will favor the generation of so-called SERS active sites “hot spots” that will be located in surface roughness, on the edges of nanotubes and between them. Therefore, the obtained nanotopography in accordance to the electromagnetic theory will effectively enhance Raman spectra on a rough surface composed of a small plasmonic metal nanoparticles below 100 nm.^6^

**Figure 1:**
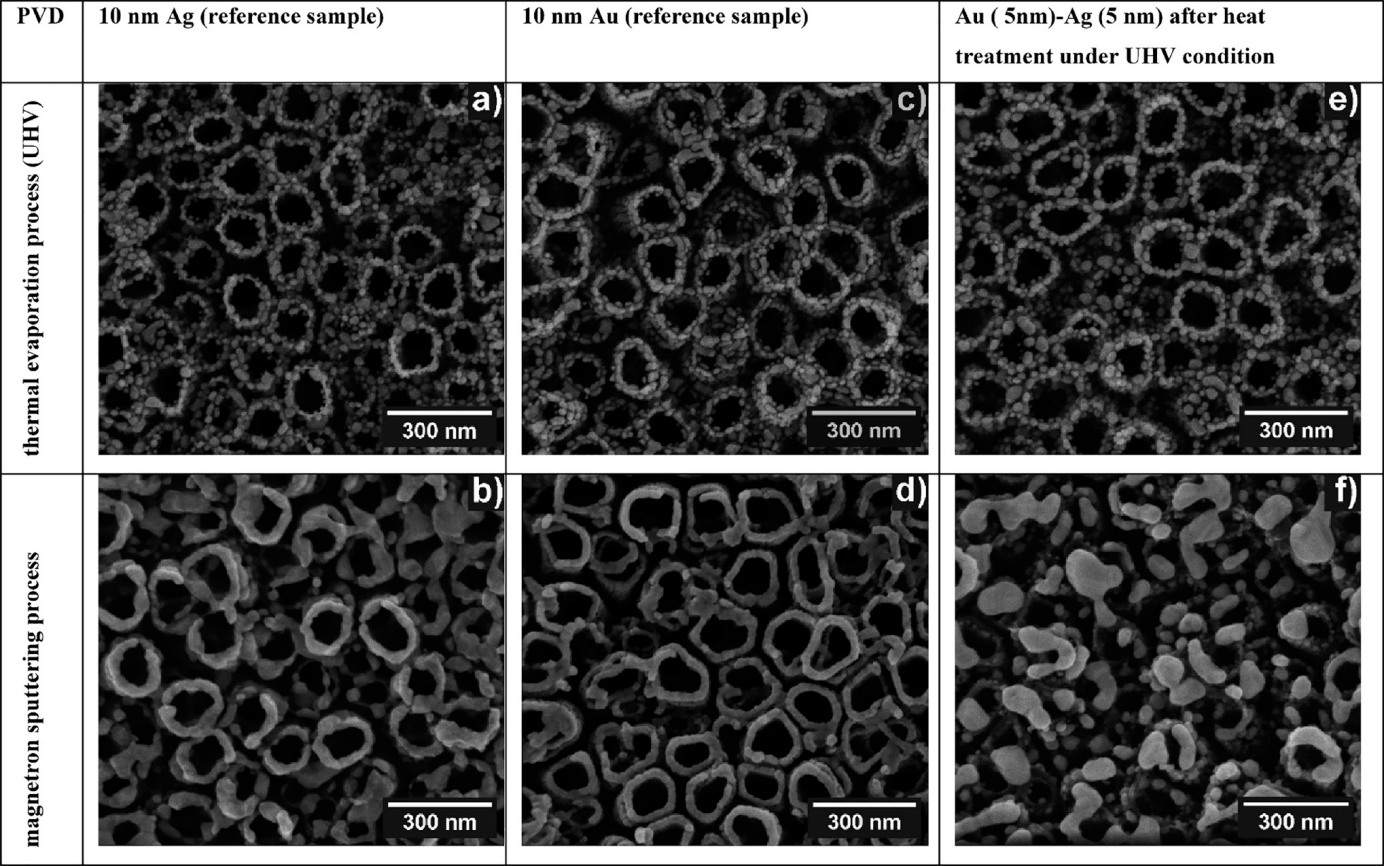
Surface morphology of TiO_2_ (25 V) nanotubes with Ag (a, b) and Au (c, d) monolayers after two different sputtering processes, with a thickness of ∼10 nm (thermal evaporation method or magnetron sputtering), and after a two-stage annealing process under UHV conditions of the systems layer by layer (5 nm Au/5 nm Ag) (e, f).

Our further research was focused on determining the structure of the resulting thin metallic layers, especially after annealing. The XRD spectra revealed strong signals only from Ti and anatase. The anatase phase appeared as a result of the thermal treatment. No characteristic signals from Au and Ag were observed.^28^ This result suggests that the thickness of the metallic layers formed on the porous TiO_2_ substrate was too thin for standard XRD measurements. Moreover, the degree of X-ray radiation dispersion in the oxide matrix significantly limited the possibility of detecting signals from Au and Ag. The proximity of the characteristic spectral lines for the Au–Ag alloy to the strong signals from Ti and TiO_2_ had a negative impact on the structural identification of the analyzed layers, which is clearly visible in Figure S1 (Supporting Information). Therefore, high-resolution STEM observations were used to determine the structure of the layers obtained after deposition and heat treatment under UHV conditions. For this purpose, cross sections of samples previously prepared using the FIB (Focus Ion Beam) method were used. Thanks to such preparation, the distribution of plasmonic metals on the surface of the nanotubes, and along the main axis of their formation, could be visualized. High-resolution observations revealed that, in the case of the sample prepared under UHV conditions (thermal evaporation of metals layer by layer + two-stage annealing), the metals were located mainly on the tops of the nanotubes decorating the surface. However, it can be seen that some metal nanoparticles penetrated into deeper regions of the nanotubes, and some are visible even at the bottom (Figure 2a). This distribution was possible because the average diameter of the nanotubes is ∼110 nm,^27^ much larger than the size of the nanoparticles being sputtered. Our previous studies of Au/TiO_2_ systems in photoelectrocatalytic applications have shown that the average size of the Au nanoparticles obtained by the same method as in the current work was 5.1 ± 2.6 nm.^29^ This means that, in the initial phase of sputtering, Au nanoparticles can penetrate deep into the nanotubes. This is also possible because of the constant evaporation rate of ∼0.03 nm/min of the Au deposited first. As can be seen, the metal deposition process was carried out very slowly under UHV conditions, making it possible to control nanoparticle size. The Au nanoparticles first decorated the nanotubes’ edges/walls, reducing their size over time. This probably affected the sputtering of the Ag nanoparticles, which had a more limited access to the nanotube walls, causing silver to be mainly located on the Au surface. The substrates prepared in this way were then annealed using a two-stage thermal treatment in UHV conditions: 300 °C/8 h + 450 °C/0.5 h,^20^ Figure 2a. The result of such processing was the appearance of larger clusters/agglomerates of nanoparticles. On the other hand, spherical nanoparticles could be seen inside the nanotubes, and did not form such objects. A completely different distribution was obtained for the sample sputtered by magnetron sputtering for which the same layer-by-layer arrangement was used: 5 nm-thick Au layer + 5 nm-thick Ag layer. This sample was annealed under the same conditions as the thermally evaporated sample. Also, for this sample, distinct agglomerates of nanoparticles can be seen, which formed much larger irregularly shaped objects (Figure 2b). One has the impression that they are spreading over the edges/walls of the nanotubes. This distribution is due to the substrate preparation method, where magnetron sputtering typically leads to the rapid formation of “flat metallic structures” at a sputtering rate of 0.15 nm/s. Then, the nanoparticles are located mainly on the edges and walls, directly at the surface of the TiO_2_ NTs, without penetrating deeper, as evidenced in the case of the thermally evaporated samples. Second, such a process probably led to a much faster effect of reducing the diameter of the nanotubes. As a result, a layer-by-layer system was obtained - Au/Ag, and nanoparticles from individual metals had a lower chance of interpenetrating each other, as they did in the case of the thermally evaporated samples. High-resolution observations of the deposits annealed in UHV conditions confirmed that they are metallic. The interplanar distances between the columns of atoms with a high degree of order can be observed in Figure S2 (Supporting Information). The lattice parameter was determined based on a Fast Fourier Transform (FFT) of the HR-TEM images to visualize the diffraction patterns, where the *d*-spacing values were 0.420 Å and 0.490 Å, respectively, see Figure S3 (Supporting Information). The data recorded may suggest the formation of an Au–Ag alloy with an fcc structure.^30–32^ Similar SAED images were obtained for the Au–Ag alloys formed by chemical methods in an exchange reaction.^32^ Nevertheless, it should be noted here that the diffraction patterns for Ag, Au, and the Au–Ag alloy are all similar to each other due to their almost identical lattice parameter. Srnova-Sloufova and coauthors determined that the lattice constant calculated from diffraction rings without alloy calibration differs from the Au and Ag calibrations by less than 1%. This makes the registered diffraction pattern difficult to solve.^31^

**Figure 2:**
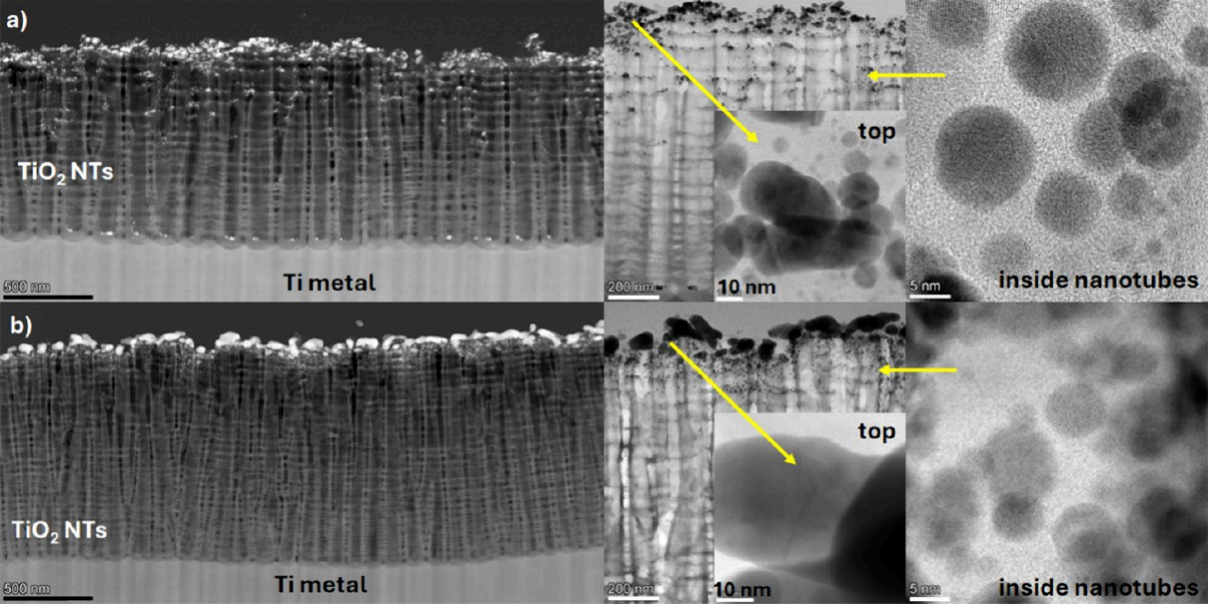
Cross section of TiO_2_ NTs bimetallic deposits after the two-stage annealing process under UHV conditions for thermally evaporated (a) and magnetron-sputtered (b) samples. The cross sections show the distribution of metals on the surface and inside the nanotubes, along with the nanoparticles’ size and structure.

To confirm the above assumptions, a chemical composition analysis was performed on cross sections of the tested materials, using the EDX method. An analysis of the thinned samples provided valuable information in the nanoareas. Figure 3 shows distribution maps of Ag, Au, Ti, and O elements for substrates after thermal vapor deposition under UHV conditions (a) and after magnetron sputtering (b). Comparing the recorded EDX maps of the produced materials, it can be clearly seen that

- first, the images of the spectral lines AuM_α_ (2.123 kV) and AgL_α_ (2.983 kV) correspond to each other and are consistent with the position of the metal nanoparticles on the surface and inside the nanotubes in the near-surface zone,
- second, due to the specificity of the metal deposition process, their amount in the near-surface zone is more significant in the sample prepared using the magnetron sputtering method,
- third, the images of the spectral lines of TiK_α_ (4.508 kV) and OL_α_ (0.525 kV) overlap, which means that the metallic layers are deposited on a titanium oxide substrate.

**Figure 3:**
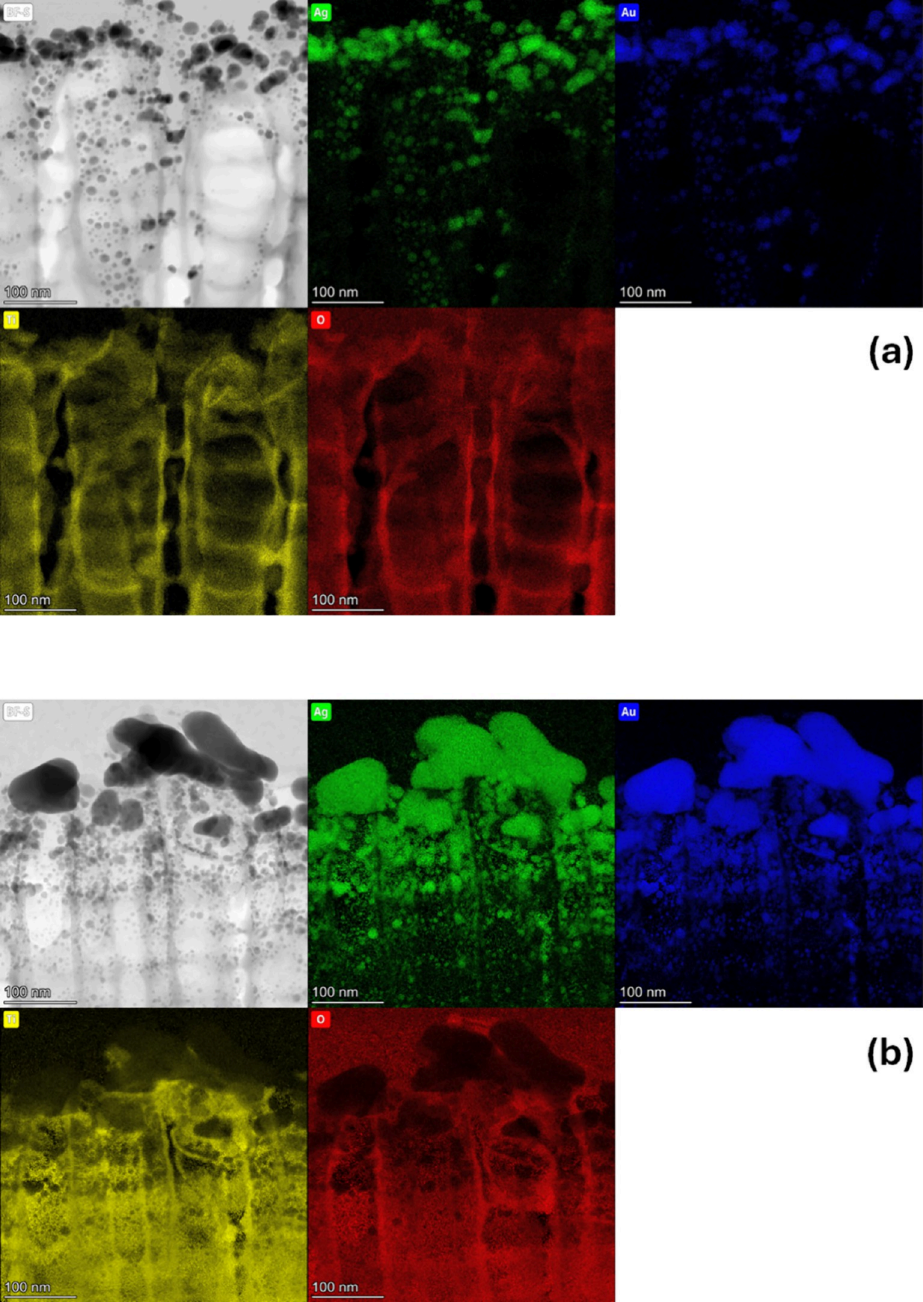
EDX chemical composition maps of the obtained bimetallic layers on TiO_2_ NTs after the two-step annealing process under UHV conditions: thermally evaporated sample (a) and magnetron sputtering sample (b).

Further analysis using the EDX method focused on recording local spectra in selected areas of the samples. The percentage of Au and Ag in the alloy layer was determined based on these. To determine the composition of the alloy as precisely as possible, the results of the EDX analysis were normalized only to the share of these two elements. This procedure was intended to limit the influence of the oxide substrate on the result of the quantitative analysis, because the excitation energies for OK_α_ (0.525 kV) and TiL_α_ (0.452 kV) differ by 73 eV, taking into account the resolution of the energy dispersion analyzer used at a level of ∼120 eV. Table 1 shows the atomic % of Au and Ag in the alloy. The results are pretty well consistent with the observations of other researchers in this field.^33,34^ On their basis, the atomic ratio of Au to Ag was determined, which varied from about 1.10 to about 1.35, depending on the area of analysis: surface or cross-section.

**Table 1:** Normalized Chemical Composition of the Alloys Produced on the Surface of TiO_2_ Nanotubes Expressed in At. %

**PVD**		**Au At. % (EDX)**	**Ag At. % (EDX)**	**Au:Ag atomic ratio (EDX)**
thermal evaporation (UHV)	SEM (top)	52.3	47.7	1.10
	STEM (cross-section)	57.4	42.6	1.35
magnetron sputtering	SEM (top)	56.4	43.6	1.29
	STEM (cross-section)	55.4	44.6	1.24

The chemical composition of the materials produced was additionally checked using the XPS method. The XPS spectra for 10 nm thick gold and silver monometallic layers on the surface of TiO_2_ nanotubes as reference materials are shown in Figure S4 (Supporting Information). XPS analysis confirmed the presence of Au and Ag in the metallic form, where individual binding energies (BE) were assigned to the 84.0 and 368.2 eV, respectively. Characteristic bonds for Ti with O were also identified, which correspond to the position of titanium dioxide at an energy of 459.3 eV for Au and Ag monometallic layers. Deconvolution of the O 1s peak also revealed that the main maximum can be related to the Ti–O bond in the TiO_2_ lattice at the BE energy around 530.5 eV. While, Figure 4 shows typical HR-XPS spectra recorded from the surface of a sample prepared under UHV conditions: metal deposition + two-stage thermal treatment. Applying our combined vacuum system, it was possible to perform an *in situ* XPS analysis immediately after both nanotube surface modification processes. The resulting spectra confirmed also the presence of Au (a) and Ag (b). The determined binding energies (BEs) for the main maxima of both elements can be assigned to the metallic state: Au 4f_7/2_–84.1 eV,^35^ Ag 3d_5/2_–368.1 eV.^35^ At higher binding energy values, Me–O bonds can be observed, which are probably the result of thermal treatment despite the vacuum conditions used.^36,37^ Moreover, the shown Ti 2p spectrum suggests the presence of Ti–O type bonds in the sample, where the peak at 459.4 eV corresponds to Ti^4+^ in the TiO_2_ lattice (c).^29^ This is also indicated by the maximum of the oxygen signal O 1s at the BE of 530.6 eV, which is responsible for the presence of metal oxides in the sample, where Me-O bonds are mainly assigned to Ti–O (Figure 4d). The remaining maxima are typical surface impurities in the form of carbon and oxygen functional groups (see Figure 4d,e). Their presence is also indicated by the distribution of the carbon peak C 1s, where individual maxima above 285.5 eV can be assigned to C–O, and C=O bonds, respectively.^38,39^ Additionally, the Au/Ag ratio was determined to be 1.01. This is an expected value, because the amount of gold and silver deposited was the same. The discrepancy between the EDX and XPS results is probably due to the depth resolution of the techniques used, where the XPS method is characterized by information in the order of several nm,^38^ thanks to which only the alloy layer shown in Figure 3a,b was analyzed. Taking into account the different distribution of metals inside the nanotubes, as shown in Figure 2, the EDX method made it possible to determine the chemical composition in a more average and volumetric manner.^40^ The XPS-VB spectra are also an interesting result. They may indicate the possibility of forming an Au–Ag alloy on the surface of TiO_2_ nanotubes. Such spectra are sensitive to changes in the electronic structure of the materials produced. After a two-stage thermal treatment, the XPS-VB band (Figure 4f) has an intermediate shape in relation to the Au monolayer or Ag deposited on nanotubes using the same technique. Then, the characteristic peak maxima can be assigned to Au and Ag. This peak shape has a set of unique features that can only be associated with the presence of an Au–Ag bimetallic system on the TiO_2_ nanotubes, which is consistent with our previous observations.^28^

**Figure 4:**
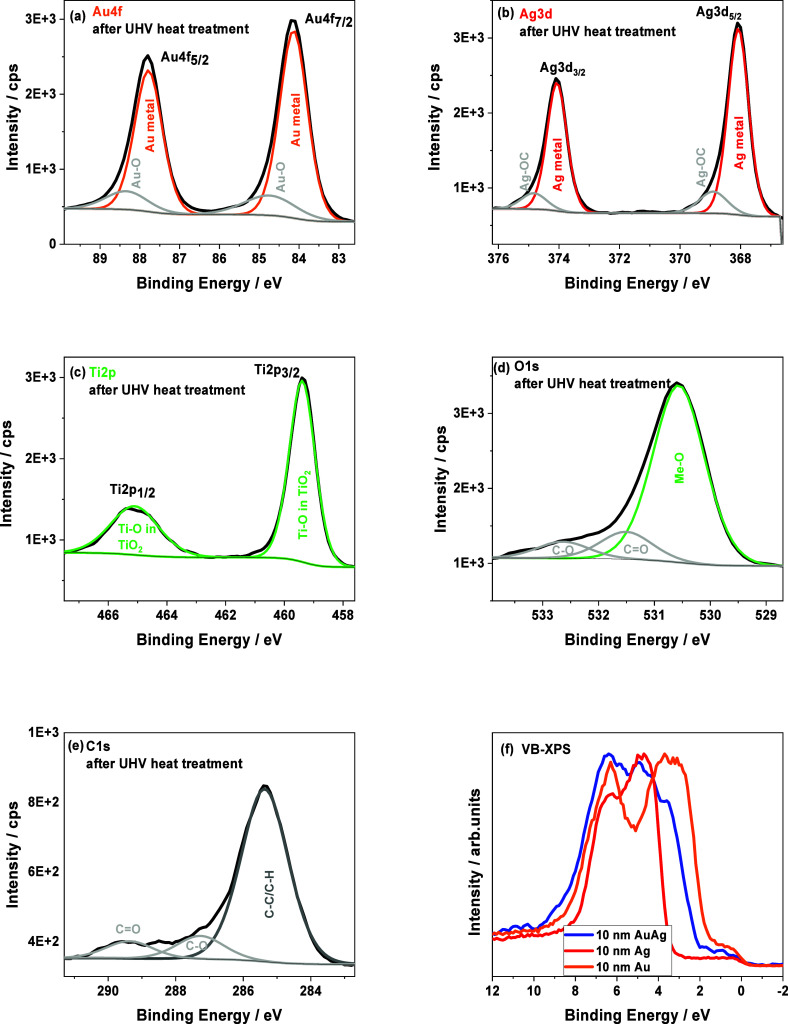
Examples of high-resolution XPS spectra recorded *in situ* after thermal evaporation of plasmonic metals and a two-step annealing process under UHV conditions (a, b, c). XPS-VB spectra for gold and silver monolayers were compared to standards of pure metals and the Au–Ag alloy system (d, e, f).

After a detailed material characterization of the oxide substrates with a thin layer of plasmonic metals produced, further studies were carried out regarding their use as active SERS platforms for detecting vitamin B12. Considering the complexity of this molecule, which forms a macrocyclic corrin system with a central Co atom, it was initially proposed to use a probe molecule of 4-mercaptobenzoic acid (PMBA). This molecule type is often used as a marker in biological applications.^41^ We applied it to determine the enhancement factor of the measured Raman spectra. The spectra of PMBA are dominated by two characteristic bands, which were attributed to the vibration modes of the aromatic rings at about 1590 cm^–1^ (ν_8a_) and 1080 cm^–1^ (ν_12_)^42^ The amplification of these bands by plasmonic metals such as Au and Ag has been described in detail by other researchers, and is consistent with our own observations.^42,43^ Nevertheless, at this point it should be added that the highest enhancement was obtained for silver monolayers with a thickness of 10 nm, and the lowest for Au monolayers of the same thickness, regardless of the metal deposition method used. Indirect enhancement was obtained for 5 nm Au/5 nm Ag bimetallic layers after a two-stage thermal treatment leading to the formation of an alloy under UHV conditions (see Figure 5a,b). The observed decrease in the intensity of the Raman spectra of PMBA for alloyed systems is related to the change in the plasmonic properties of such materials. It is known that samples consisting only of Ag nanoparticles usually have a much higher enhancement factor than Au and AuAg alloy, as shown in the Figure 5. This effect was already well recognized in the early 1980s by Pettinger and Wetzel.^5^ Adding Au to Ag suppresses the strengthening, but thanks to the formation of an alloy system on the surface of TiO_2_ nanotubes, a stable substrate was obtained in terms of chemical properties (AuAg/TiO_2_), which is characterized by intermediate enhancement between Ag and Au. It should also be noted that the average intensity from the recorded acid spectra differs significantly depending on the sample preparation method. This difference is very beneficial for substrates thermally evaporated in UHV conditions.

**Figure 5:**
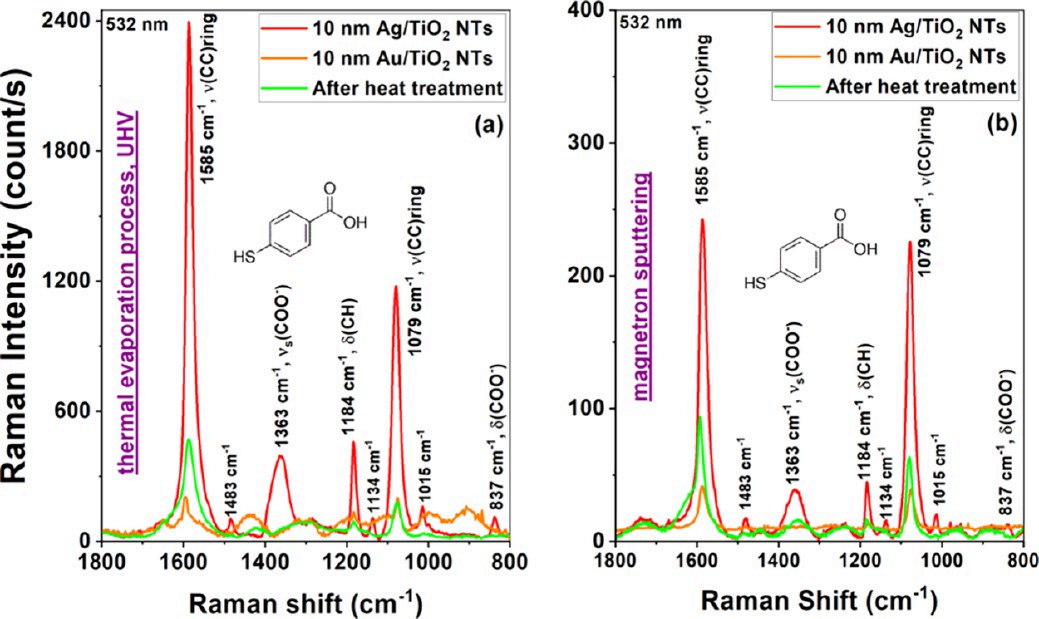
SERS spectra of 4-markaptobenzoic acid recorded on the TiO_2_ NTs with bimetallic Au–Ag layers obtained by the thermal evaporation method (a) and magnetron sputtering (b). Red spectra represent the reference sample obtained by deposition of 10 nm Ag, orange spectra correspond to the reference sample obtained by deposition of 10 nm Au, and green spectra represent the Au–Ag alloy obtained by sequential deposition of 5 nm Au and 5 nm Ag followed by the heat treatment in an ultra high vacuum.

The changes observed in the intensity of the measured SERS spectra of PMBA acid, depending on the sample preparation method, inspired us to compare the determined enhancement factors in terms of their distribution on a square surface with an edge length of 50 μm, on which 400 local spectra were measured. Figure 6 shows an *E*_F_ map distribution, where local spectra were recorded in linear sequences of 20 measurements each, with a step of 2.6 μm. For each point, the *E*_F_ factor was determined based on eq 1, implemented for our purposes from a publication Krajczewski et al.^44^

$$E_{F}=\frac{I_{\mathrm{SERS}}/N_{\mathrm{SERS}}}{I_{\mathrm{normal}}/N_{\mathrm{normal}}}$$ 1

where *I*_SERS_ is the SERS intensity, *N*_SERS_ is the number of molecules adsorbed on the plasmonic metal surface in the SERS excitation area, *I*_normal_ is the non-SERS intensity on the Pt reference surface, and *N*_normal_ is the number of molecules in the non-SERS experiments on Pt.

**Figure 6:**
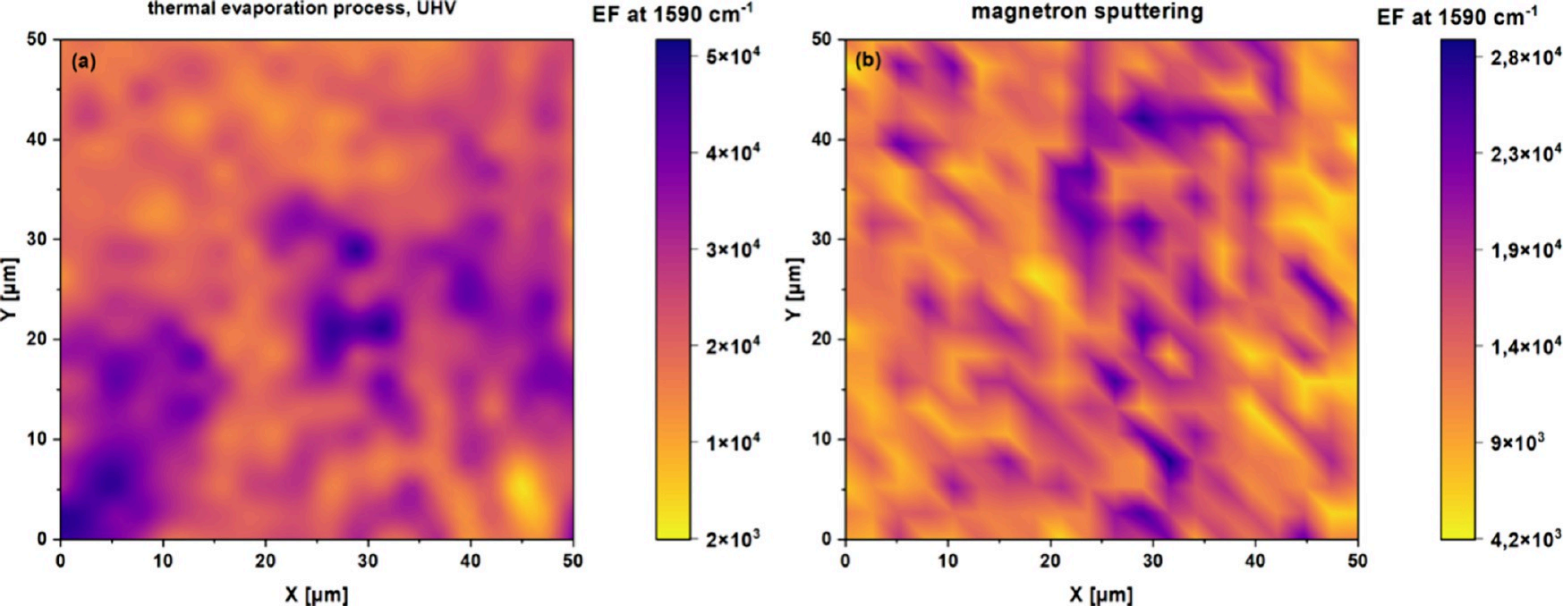
Distribution map of the enhancement factor on the surface of samples with bimetallic Au and Ag layers obtained using the thermal evaporation method (a) and magnetron sputtering (b) after two-stage annealing under UHV conditions. The *E*_F_ was estimated based on locally collected SERS spectra recorded every 2.6 μm, line by line, from a 50 μm × 50 μm region.

This approach allowed those authors to visualize how the Raman signal amplification changes from site to site due to the different distributions of plasmonic metals, in the form of an alloy, on the ordered structure of TiO_2_ nanotubes. In the case of thermal evaporation under UHV conditions (Figure 6a), the tested surface was characterized by a more favorable distribution of the *E*_F_ factor. One can see more areas that effectively enhance the Raman spectra of PMBA compared to the magnetron-sputtered substrate (Figure 6b). In addition, the adopted color scale of the *E*_F_ parameter adopted changes more monotonously than in the magnetron sample, for which much more significant contrast changes are visible. This means that the change in *E*_F_ is closely correlated to the formation of ‘nano objects’ on the surface of the nanotubes after the UHV thermal treatment, as shown in Figure 3. The large, irregular shapes of the remelted objects are less conducive to generating strong amplification than a surface characterized by a more homogeneous distribution in the form of spherical nanoparticles, where their average size was 16 ± 7 nm.^28^ This kind of surface promoted the formation of a greater number of “hot spots”, which are generated from two processes: thermal evaporation, and two-stage annealing in UHV conditions (10^–8^ mbar). Also, this solution first led to the formation of nanoparticles, layer by layer, smaller in size than those produced during magnetron sputtering. Second, this method controlled the growth of particles during annealing, leading to a more ordered metallic nanostructure on the TiO_2_ NTs surface, where the distances and gaps between the nanoparticles were more regular than in the case of the substrate obtained by magnetron sputtering. Subsequently, SERS tests were carried out on such defined substrates using the probe molecule–vitamin B12 at various concentrations.

In the last few decades, several attempts have been made to use SERS spectroscopy to detect various B vitamins, which include B1 (thiamine, aneurine), B2 (riboflavin), B3 (niacin–nicotinic acid and nicotinamide), B6 (pyridine derivatives: pyridoxine, pyridoxal and pyridoxamine and their 5′-phosphates), B7 (biotin), B9 (folic acid, folate), B12 (cobalamin, cyanocobalamin). An interesting example is the analysis of *p*-aminobenzoic acid (PABA) in vitamin B complex dissolved in ethanol using a silver-coated alumina-based substrate, where PABA (vitamin B10) was used as a marker because of its characteristic structure and because it can be selectively identified by SERS.^45^ The same group of researchers, using the same type of platform, studied the dependence of the intensity of the SERS signal as a function of the concentration of nicotinamide (vitamin B3) to detect this compound at the ppm level.^46^ Another example was the use of a colloidal system with silver nanoparticles for the detection of vitamin B12 in an aqueous solution and atmosphere, where the effect of water on the structure of vitamin B12 as a result of its interaction with Ag at low laser power was investigated.^21^ Ag-coated TiO_2_ nanotubes were also used to detect vitamin B12, where Jafari and coauthors demonstrated the possibility of detecting vitamin B12 at a level of 10^–8^ M in an aqueous solution while maintaining the high measurement repeatability of the system. They also found that the designed substrate has a self-cleaning effect and can be reused for four consecutive cycles without a noticeable loss in SERS efficiency.^24^ Kokaislová and Matějka investigated the adsorption mechanism of various B vitamins (B2, B3, B6, B9) on a surface of gold or silver. They checked how this mechanism affects the intensity of measured SERS spectra, and their repeatability.^47^ In terms of the practical application of the SERS method, Radu et al. proposed using this technique in food analysis to detect vitamin B2 and B12 based on appropriate protocols and comparative markers.^23^ Junior et al. analyzed the field of quantitative determination of B vitamins (B1, B2, B3) in pharmaceutical samples using a colloid gold substrate.^25^ These various attempts to use the SERS method to analyze B vitamins were determined by the latter’s characteristic structure. Vitamin B1 is a heterocyclic chemical compound composed of thiazole and pyrimidine rings connected by a methylene bridge. Vitamin B2 is a combination of ribitol and flavin, and vitamin B3 is a combination of nicotinic acid and nicotinamide. Vitamin B5 is a compound of pantothenic acid and its derivatives, while vitamin B6 is a group of 6 organic chemical compounds, pyridine derivatives: pyridoxine, pyridoxal and pyridoxamine and their 5′-phosphates. Vitamin B7 is a heterocyclic organic chemical compound that contains a system of condensed rings–imidazolidine and thiolanic with an alkyl chain ending in a carboxyl group. Vitamin B9 is folic acid, and vitamin B12 is cobalamin, which contains cobalt as its central atom–a strong pigment.^23,26,47^ These conditions have led to special attention to vitamin B12, which plays an important role in many biological processes of the body: it supports the health of the nervous system, participates in the production of DNA, participates in the metabolism of fatty acids and amino acids, affects the production of red blood cells, supports the health of the skin and mucous membranes, and affects the metabolism of homocysteine.^48^ Considering these factors, and the fact that researchers have so far mainly used Ag or Au monometallic layers in SERS substrates, this time a bimetallic Au–Ag system was proposed. Au–Ag bimetallic nanostructures of various sizes and shapes have attracted great attention from investigators due to the unique optical properties they possess compared to their pure element counterparts. According to electromagnetic theory, Ag is more effective than Au for plasmonic enhancement, while Au provides greater chemical stability.^49^ This is important for measurements carried out in aqueous solutions, where Ag degradation processes can progress faster than those of Au–which can lead to a decrease in the intensity of the measured SERS spectra and in the stability of the substrate. Therefore, Au–Ag nanostructures’ trade-off in physical and chemical properties can benefit this application.^15^ Figure 7 shows the SERS spectra of vitamin B12 on the substrates produced after a two-stage heat treatment under UHV conditions. For both substrates, after different processes of deposition of bimetallic Au–Ag layers a characteristic band can be observed at about 1600 cm^–1^, which can be attributed to the corrin ring, i.e., the “core” of vitamin B12 (cobalamin).^21^ Comparing the intensity of the recorded SERS spectra, it can be seen that it is much higher for the UHV thermally evaporated samples than the magnetron-sputtered samples. This observation confirms our previous observations for PMBA acid. The amplification of the SERS signal is related to the distribution of the *E*_F_ factor in the context of morphological changes induced by the thermal treatment and the appearance of Au–Ag alloy systems on the surface of the TiO_2_ nanotubes. At this point, it should also be noted that a change in the concentration of vitamin B12 in an aqueous solution causes a decrease in the intensity of the measured spectra. Jafari et al. observed for an Ag/TiO_2_ system that, as a result of changing the logarithm of vitamin B12 concentration in a range of from 10^–5^ M to 10^–8^ M, the intensity of SERS spectra decreased linearly, and the determined correlation coefficient was 0.9747 (parameter R^2^).^24^ A very close result was obtained for the sample thermally evaporated under UHV conditions, where the coefficient factor was determined to be 0.9068, see Figure S5 (Supporting Information). A much worse correlation was obtained for the magnetron sputtering sample, where R^2^ was 0.6507.

**Figure 7:**
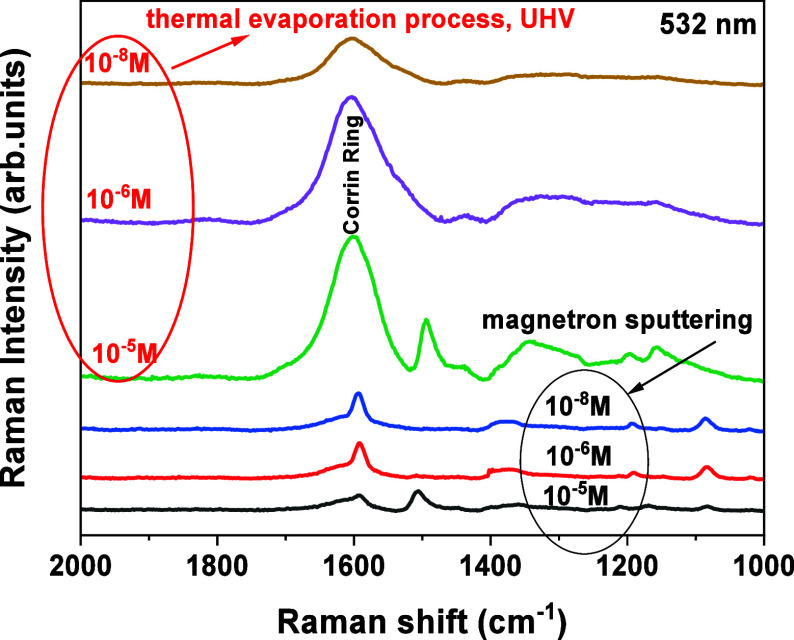
Average spectra from 400 measurements on the surface of TiO_2_ substrates modified by PVD methods for vitamin B12 at concentrations ranging from 10^–5^ to 10^–8^ M.

These results significantly affected the stability of the measured SERS signal for vitamin B12 on the surface of the two-stage annealed samples, as shown in Figure 8. Depending on the deposition method of Au and Ag on the surface of TiO_2_ nanotubes, it can be seen that the thermally evaporated sample under UHV conditions shows better measurement stability than the magnetron-sputtered sample. The dispersion of the results is much smaller for it (red points), and amounts to 3.93% (RSD) and 14.99% (RSD) for the magnetron sample (black points). The measurements were taken at a vitamin concentration of 10^–5^ M. Because the correlation coefficients for the change in vitamin B12 concentration and the stability of the SERS signal for the characteristic corrin band for the sample thermally evaporated in a high vacuum are much better than for the magnetron sample, this leads to the conclusion that the method of substrate preparation has a huge impact on efficiency. This is because SERS spectroscopy is based on measuring the Raman scattering radiation of molecules adsorbed on plasmonic metal surfaces, which results in a significant enhancement of the measured SERS signal compared to the classical Raman measurement.^50^ Typically, the most significant enhancement is obtained when the plasmon frequency ω_p_ of the metal is in resonance with the incident radiation.^7,51^ Then the vibrations normal to the surface are amplified most strongly. The best morphology for surface plasmon resonance, therefore, is small particles of these metals below 100 nm, or their agglomerates.^52^ In our case, such a structure was obtained for the Au–Ag sample produced under UHV conditions. Thanks to an appropriate distribution of plasmonic metal nanoparticles on the surface of the nanotubes and in their interior, a strong SERS effect was obtained, which was highly stable regardless of the measurement location on the sample, in contrast to the magnetron-sputtered sample. Despite some similarities between both substrates, the size, shape, and organization of Au–Ag nanostructures around the tops of the TiO_2_ nanotubes turned out to be crucial in creating a strong local electromagnetic (EM) field enhancement, which is particularly important for SERS spectroscopy.^7,52,53^ Nevertheless, from a practical point of view, both the thermal evaporation in a vacuum and magnetron sputtering methods can be used to prepare active SERS substrates. Currently, the technique of preparing substrates is strongly related to their area of application. Therefore, Nag and coauthors proposed the use of SERS substrates based on Ag–Au and Ag–Cu bimetallic systems as biomedical sensors for the detection of anticancer drugs like mitoxantrone (MTO)^53^ or monitoring catalytic reactions of 4,4′-biphenyldithiol (BPDT).^54^ On the other hand, ternary Ag–Au–Cu systems were used to design active and highly sensitive platforms for detecting thiols (l-cysteine and toxic thiophenols), which is important from the point of view of environmental protection and health.^55^ In our case, thermal evaporation under UHV conditions was a better way to design active SERS platforms for detecting vitamin B12. Nevertheless, it should be noted that the substrates proposed can be universal tools for studying various molecules in analytical chemistry and beyond. This is due to the structure of the substrate itself, which is based on inert TiO_2_ nanotubes combined with a plasmonic layer with compromised optical properties and chemical stability.

**Figure 8:**
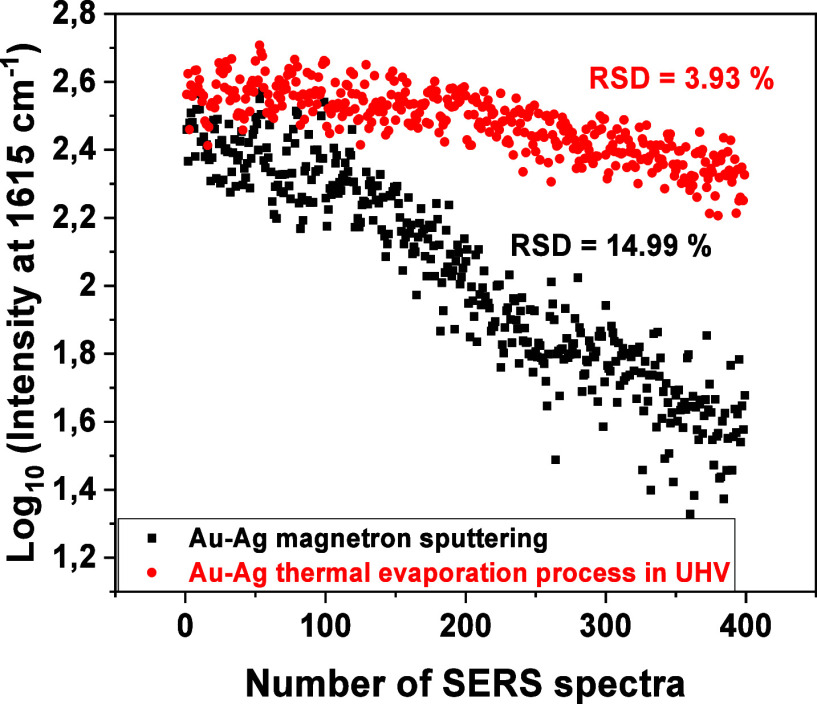
Stability of the vitamin B12 signal on the surface of plasmonic alloy layers deposited on TiO_2_ nanotubes.

## 4 Conclusions

This research showed that the thermal evaporation method under UHV conditions turned out to be more effective for producing SERS active platforms for detecting vitamin B12. In contrast to the magnetron sputtering method, this technique made it possible to deposit spherical Au and Ag nanoparticles on the walls and edges of TiO_2_ nanotubes, which, after a two-stage annealing process, transformed into larger, regularly shaped nanometric objects forming a characteristic nanotopography. This allowed Au–Ag alloyed plasmonic layers to be prepared, where the size and shape of the nanotubes determined how they were organized. This in turn led to the formation of a characteristic morphology that locally generated many places that specially enhanced the electromagnetic field, as is presented in the maps of enhancement factor distribution. Moreover, thanks to this organization of the Au–Ag bimetallic alloy on the surface of the TiO_2_ nanotubes, it was found that the average intensity of the measured vitamin B12 spectra was four times higher than in the case of the magnetron-sputtered substrates. Correlating high-resolution SEM and STEM microscopic observations with Raman measurements enabled the authors to discern the properties of the obtained layers in terms of their application as SERS active substrates.

## Supplementary Material



## Notes

Evolution of XRD spectra after various stages of surface functionalization of TiO_2_ nanotubes, HR-STEM images of the Au–Ag alloy layer, FFT images based on the HR-STEM analysis of the Au–Ag nano objects, XPS spectra for monometallic layers, SERS results for vitamin B12 (PDF)
