# Supplementary material for: Nanoplasmonic Au–Ag Alloy Coatings on the Surface of TiO2 Nanotubes for Vitamin B12 Detection by Surface-Enhanced Raman Scattering Spectroscopy
Source: ACS Omega. 2025 Jun 12;10(24):25555–68. doi: 10.1021/acsomega.5c01060 (PMC12199029; doi:10.1021/acsomega.5c01060)
Supplement: Supplementary file 1 [file ao5c01060_si_001.pdf]

Supporting information

Nanoplasmonic Au-Ag Alloy Coatings on the Surface of TiO_2 Nanotubes for Vitamin B12 Detection by Surface-Enhanced Raman Scattering Spectroscopy

Marcin Pisarek[1*], Robert Ambroziak[1], Mirosław Krawczyk[1], Marcin Hołdyński[1], Jan Krajczewski[2], Tomasz Płociński[3]

[1]Institute of Physical Chemistry, Polish Academy of Sciences, Kasprzaka 44/52, 01-224 Warsaw, Poland

[2]Faculty of Chemistry, University of Warsaw, Pasteur 1, 02-093 Warsaw, Poland

[3]Faculty of Materials Science and Engineering, Warsaw University of Technology, Woloska 141, 02-507 Warsaw, Poland

[*]corresponding author: mpisarek@ichf.edu.pl

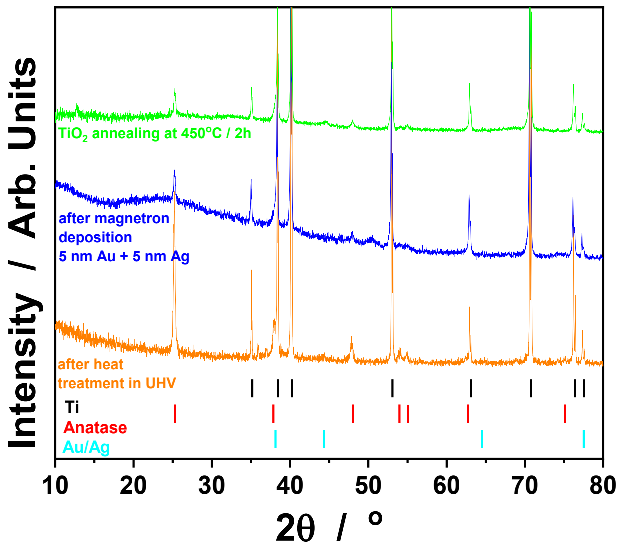

Figure S1. *Examples of XRD spectra after different stages of TiO_2 nanotube modification: after pre-annealing at 450°C in air for 2 h, after the magnetron sputtering process of a gold-silver bimetallic system (5 nm Au + 5 nm Ag), after two-stage annealing in UHV conditions (300°C / 8 h + 450°C + 0.5 h).*

A clear signal from anatase was identified as a result of pre-annealing. Immediately after magnetron sputtering and two-stage heating under UHV conditions, there are no characteristic signals from Au, Ag and the Au-Ag alloy. Similar results were obtained for a sample thermally evaporated under UHV conditions and two-stage annealing, as described in detail in our previous publication [1].

[1] J. Phys. Chem. C 2023, 127, 50, 24200–24210

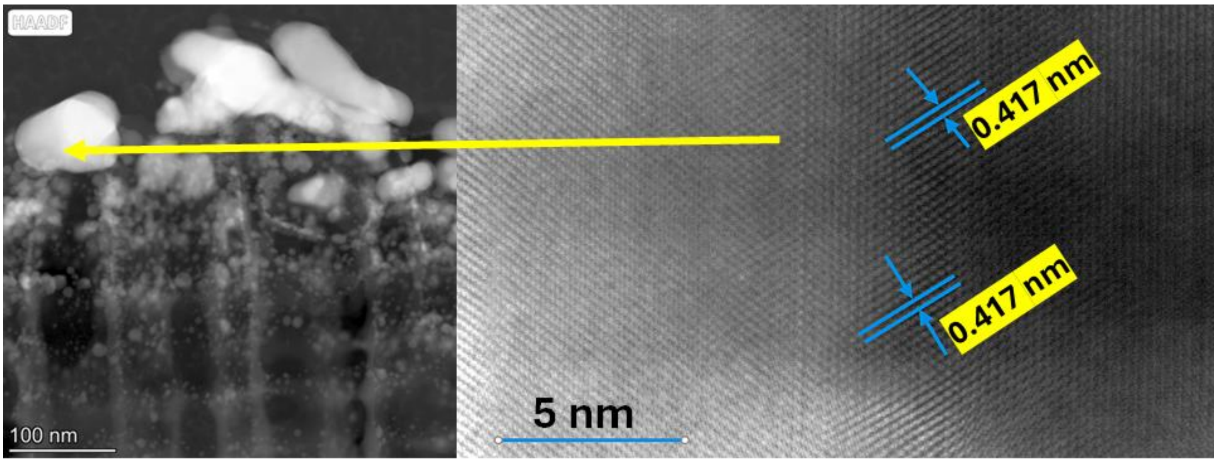

Figure S2. *Example of the HR-STEM images of the cross-section of Au-Ag nanoobjects deposited on the surface of* TiO_2 *nanotubes after a two-step UHV annealing process (magnetron sputtering sample).*

The images show the crystalline nature of the formed coatings after two-step annealing.

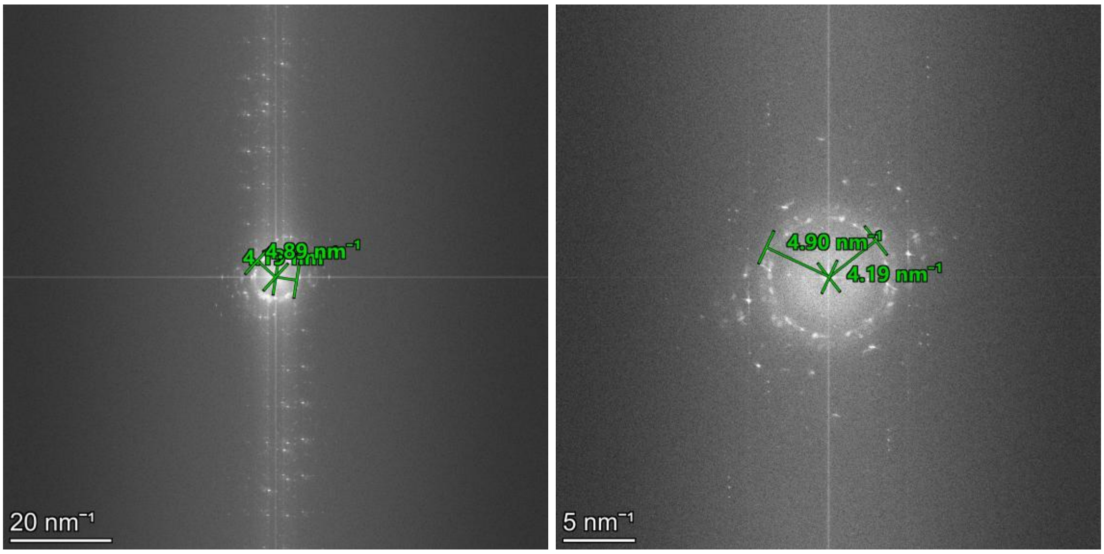

Figure S3. *Example of the Fast Fourier Transform (FFT) of the HR-TEM image, which was used for visualization of the electron diffraction pattern: metallic nanoparticles, bigger objects located at the surface of* TiO_2 *NTs, see Fig. 2S*

The diffraction images may suggest the presence of Au-Ag alloy on the surface of TiO_2 nanotubes.

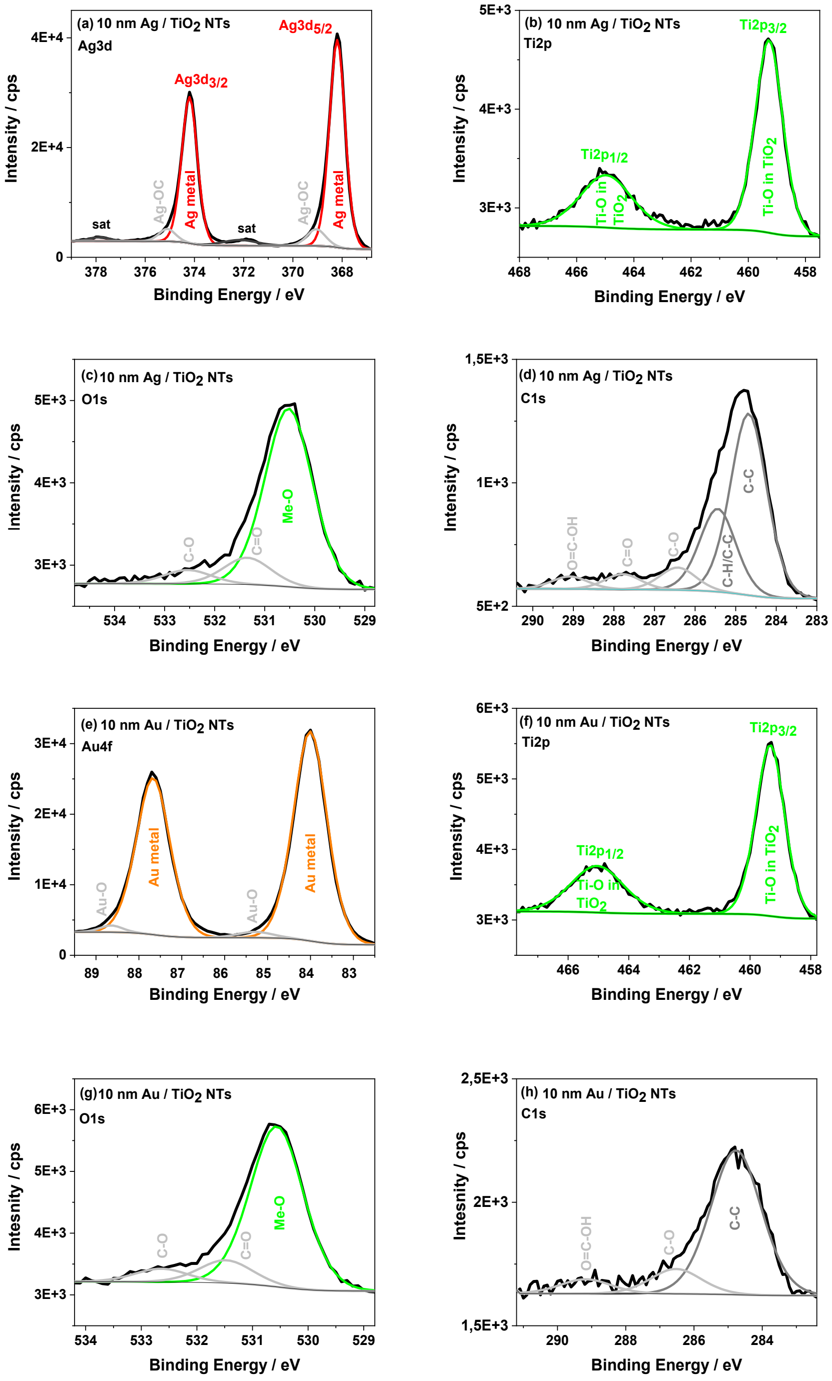

Figure S4. *A compilation of high-resolution XPS spectra for silver (Ag3d), gold (Au4f), titanium (Ti2p), oxygen (O1s) and carbon (C1s) recorded on the surface of* TiO_2 *nanotubes decorated with monometallic Ag or Au layers with a thickness of 10 nm.*

Ag and Au monometallic layers were obtained by thermal evaporation in ultra-high vacuum. Reference samples.

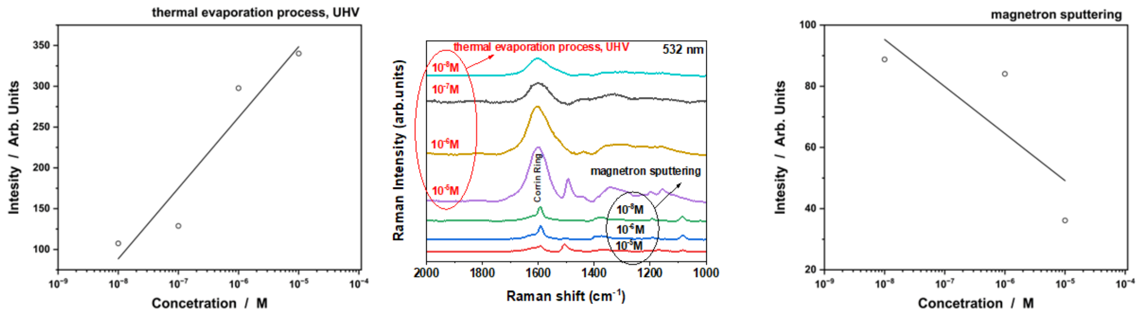

Figure S5. *Linear trend of the dependence of SERS intensities at ~1600 cm^{-1} on the logarithm concentration of vitamin B12 in water solution. Updated Figure 7 from the main MS with additional data for the sample thermally evaporated under UHV conditions.*

The change in vitamin B12 concentration in the analyzed solution is associated with a decrease in the intensity of the measured Raman spectra.
